# Supplementary material for: Metabolism of l-arabinose converges with virulence regulation to promote enteric pathogen fitness
Source: Nat Commun. 2024 May 25;15:4462. doi: 10.1038/s41467-024-48933-7 (PMC11127945; doi:10.1038/s41467-024-48933-7)

Supplementary information

Metabolism of L-arabinose converges with virulence regulation to promote enteric pathogen fitness

Curtis Cottam, Rhys T White, Lauren C Beck, Christopher J Stewart, Scott A Beatson, Elisabeth C Lowe, Rhys Grinter and James PR Connolly

Supplementary figures 1-12

Supplementary table 1 – Bacterial strains used in this study.

Supplementary table 2 – Plasmids used in this study.

Supplementary table 3 – Primers used in this study.

Supplementary references

Uncropped images

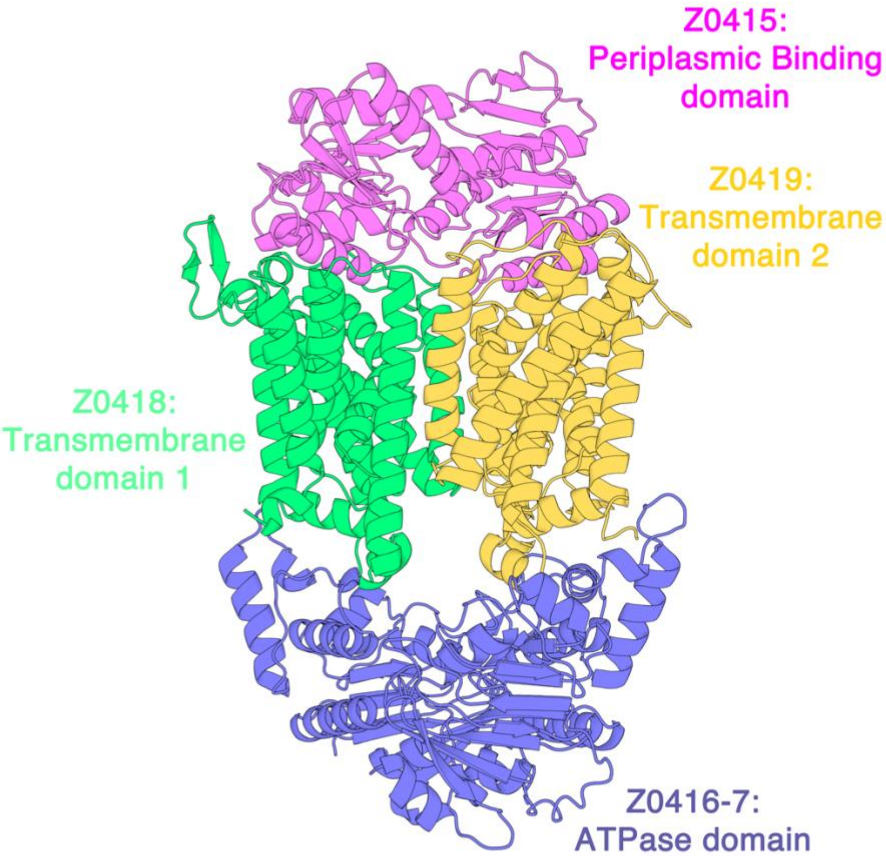

Supplementary Figure 1. Model of the Z0415-9 ABC transporter. AlphaFold2 model of the predicted Z0415-9 structure assembled at the EHEC inner membrane.

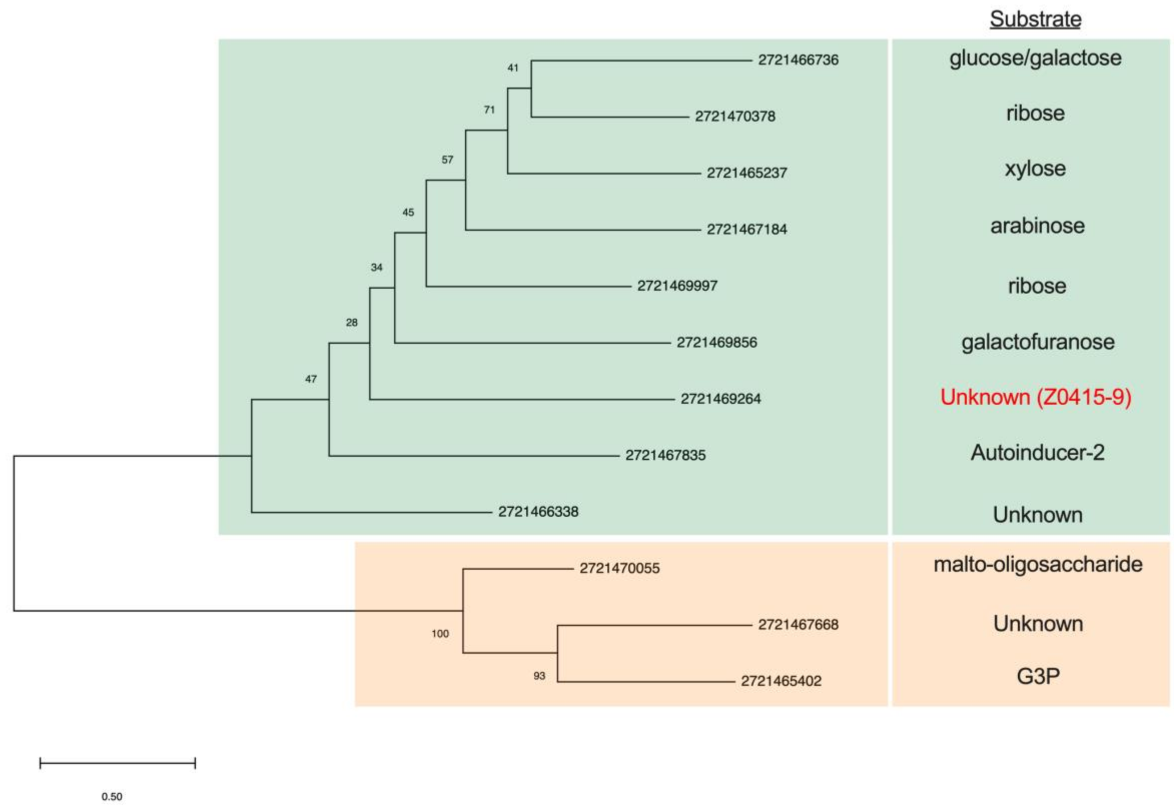

Supplementary Figure 2. Phylogenetic analysis of sugar-specific ABC transporters in EDL933. Phylogeny was inferred using the maximum-likelihood method and Le Gascuel model, with a Gamma distribution in MegaX. The tree is drawn to scale, with branch lengths measured in the number of substitutions per site. The scale bar represents 0.5 substitutions per site. Bootstrap values are indicated on the respective branches. Clades A and B are coloured green and orange respectively. The predicted substrate for each transporter is indicated next to its respective branch in the tree.

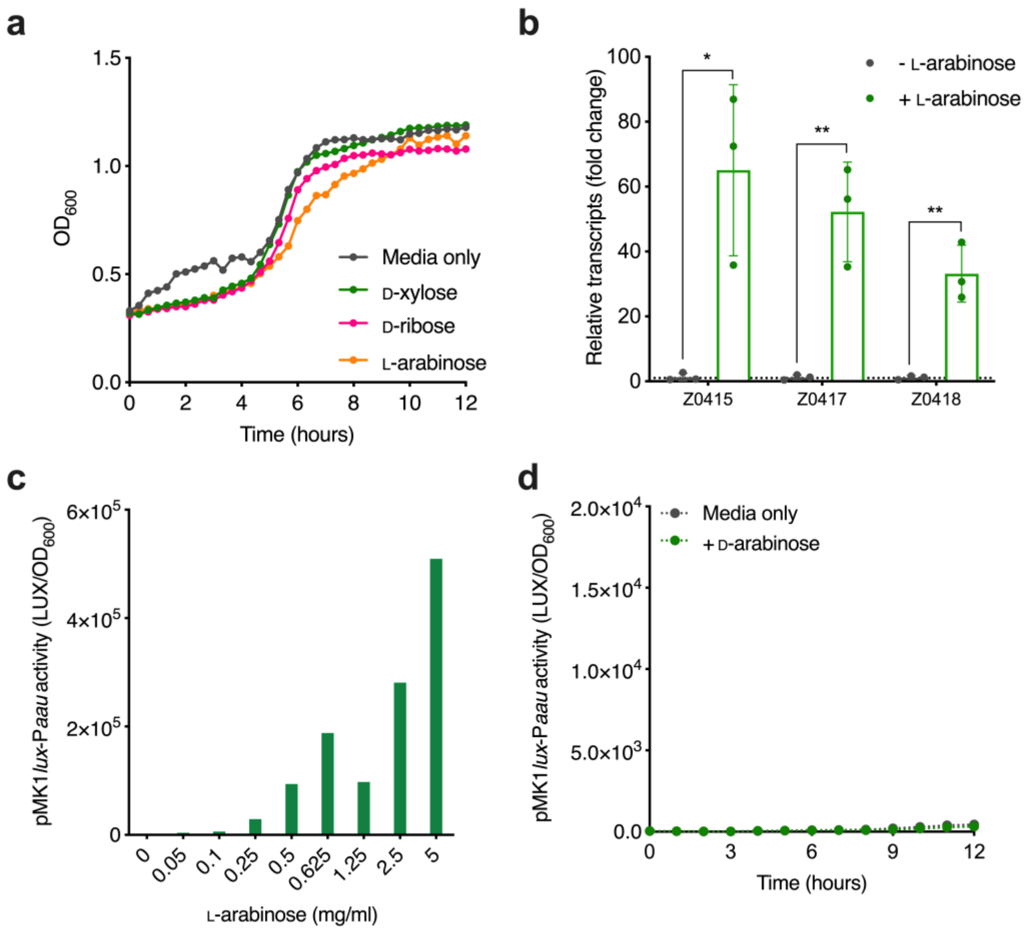

Supplementary Figure 3. The L-isomer of arabinose induces Z0415-9 in a concentration dependent manner. a, Growth curve depicting OD_{600} values (*n* = 1, repeated on three independent occasions) over time of EHEC cultured in MEM-HEPES alone or supplemented with 0.5 mg/ml of L-arabinose (green), D-ribose (pink) or D-xylose (orange). **b,** RT-qPCR analysis of Z0415/7/8 expression in RNA-derived from EHEC cultured in MEM-HEPES alone (grey) or supplemented with L-arabinose (green). The bars indicate relative fold-increase of L-arabinose treated cultures over media alone. The dotted line indicates baseline expression in comparison to the control. Statistical significance was determined by two-tailed Student's *t* test. * and ** indicate $P < 0.05$ or $P < 0.01$ respectively. Error bars represent standard deviation from *n* = 3 biological replicates. **c**, Transcriptional reporter assay of EHEC transformed with pMK1*lux*-P*aau* cultured in MEM-HEPES alone or supplemented with a range of L-arabinose concentrations. Data are depicted as luminescence units (LUX) divided by optical density (OD_{600}) of the culture after 8 hours of growth (*n* = 1). **d,** Transcriptional reporter assay of EHEC transformed with pMK1*lux*-P*aau* cultured in MEM-HEPES alone or supplemented with 0.5 mg/ml D-arabinose (*n* = 3 biological replicates).

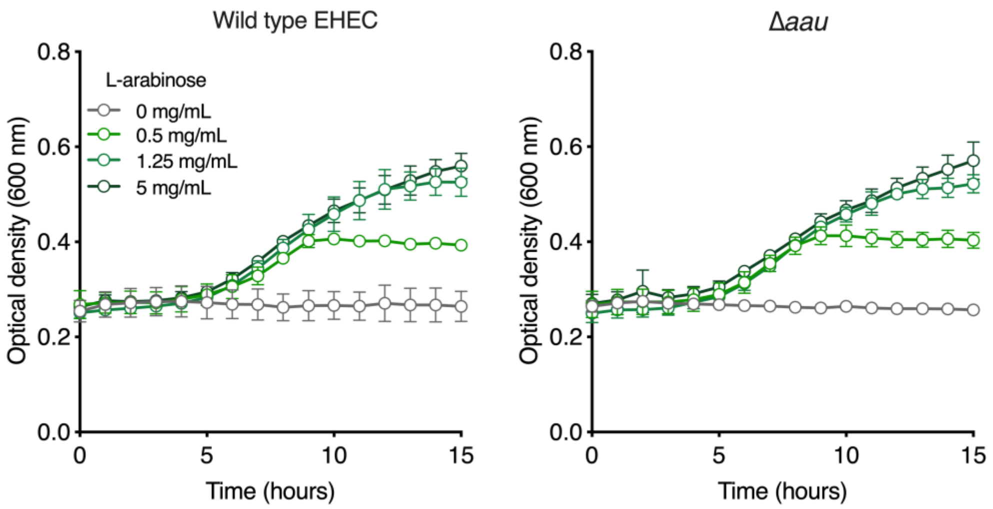

Supplementary Figure 4. Aau is not required for growth on ʟ-arabinose as a sole carbon source. Growth analysis of EHEC ZAP193 and associated Δ*aau* deletion mutant in M9 minimal media supplemented with various concentrations of ʟ-arabinose as a sole carbon source as labelled on the graph (light, medium and dark green; grey for no carbon source control). The error bars indicate standard deviation based on *n* = 3 biological replicates.

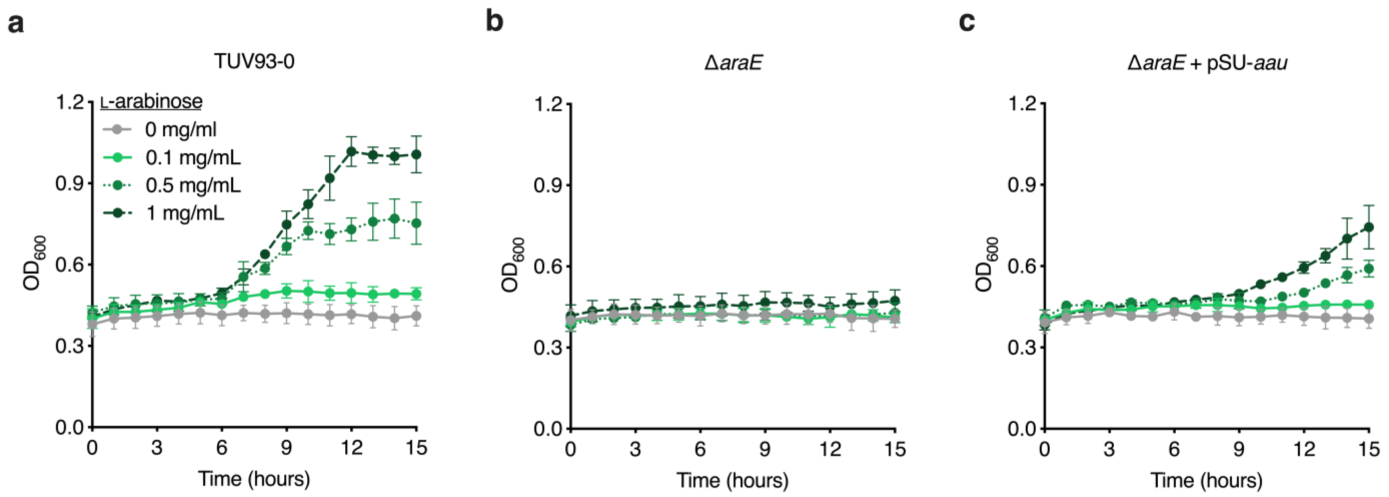

Supplementary Figure 5. Plasmid-expressed Aau enhances growth on L-arabinose in an EHEC strain with a non-functional *aau* locus. a, Growth analysis of EHEC TUV93-0 wild type in M9 minimal media containing various concentrations of L-arabinose as the sole carbon source as labelled on the graph (light, medium and dark green; grey for no carbon source control). **b,** Growth analysis of TUV93-0 Δ*araE* under the same conditions. c, Growth analysis of TUV93-0 Δ*araE* transformed with pSU-*aau* (encoding the entire *aau* locus form ZAP193) under the same conditions. Error bars represent standard deviation based on *n* = 3 biological replicates.

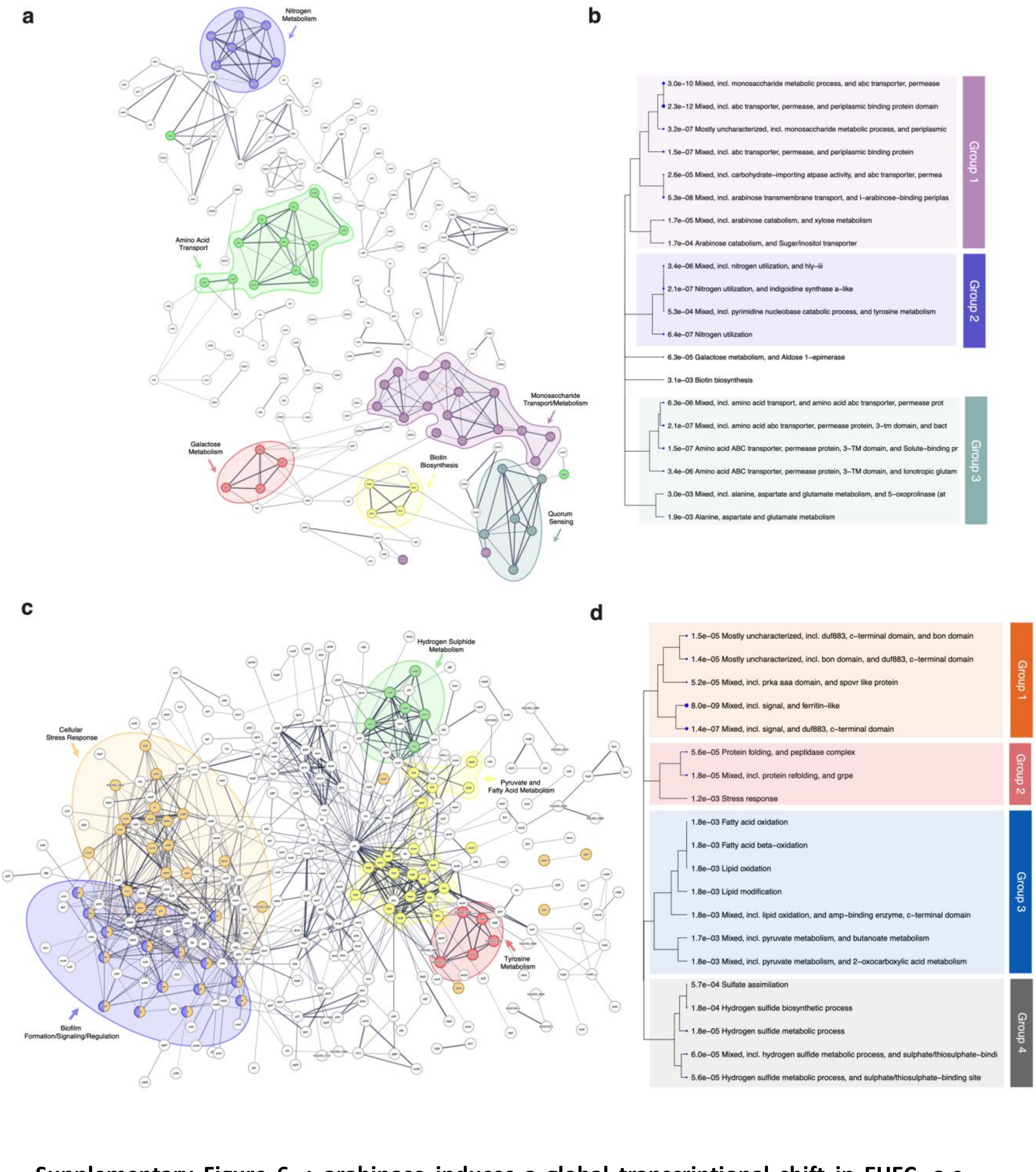

Supplementary Figure 6. ʟ-arabinose induces a global transcriptional shift in EHEC. a,c, STRING network analysis of predicted protein-protein interactions based upon upregulated or downregulated genes identified by RNA-seq analysis of EHEC cultured in MEM-HEPES supplemented with ʟ-arabinose. Line thickness is indicative of confidence in the interactions between proteins. Disconnected nodes were removed from the network to improve clarity. Distinct functional clusters that are enriched are in coloured bubbles. **b,d**, Hierarchical clustering tree summarising the correlation among significant STRING pathways generated using ShinyGO. Pathways that are enriched with shared genes are grouped together. Larger dots indicate more significant *P*-values.

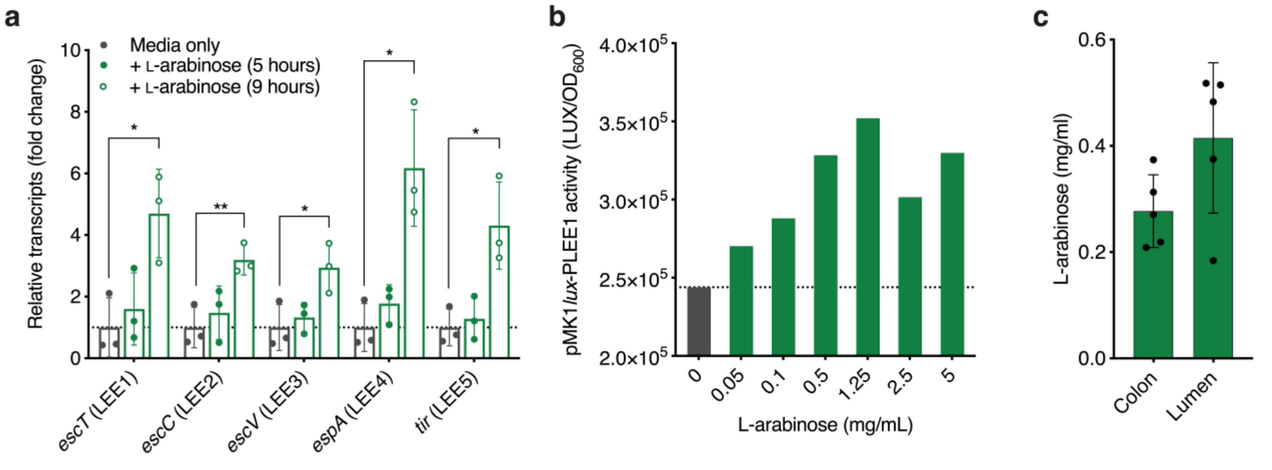

Supplementary Figure 7. ʟ-arabinose induces LEE expression at concentration relevant to the gut. a, RT-qPCR analysis of relative LEE1-5 expression in RNA-derived from EHEC cultured in MEM-HEPES alone (grey) or supplemented with ʟ-arabinose (green). The bars indicate relative fold-increase of ʟ-arabinose treated cultures over media alone. The dotted line indicates baseline expression in comparison to the control. Statistical significance was determined by two-tailed Student's *t* test. * and ** indicate $P < 0.05$ or $P < 0.01$ respectively. Error bars represent standard deviation from $n = 3$ biological replicates. **b,** Transcriptional reporter assay of EHEC transformed with pMK1*lux*-P*LEE1* cultured in MEM-HEPES alone (grey) or supplemented with a range of ʟ-arabinose concentrations (green). Data are depicted as luminescence units (LUX) divided by optical density (OD_{600}) of the culture after 8 hours of growth. The data represent a single replicate ($n = 1$) from three independent repeats of the experiment. **c,** Quantification of ʟ-arabinose from colon tissue and luminal content of BALB/c mice maintained on a conventional diet ($n = 5$).

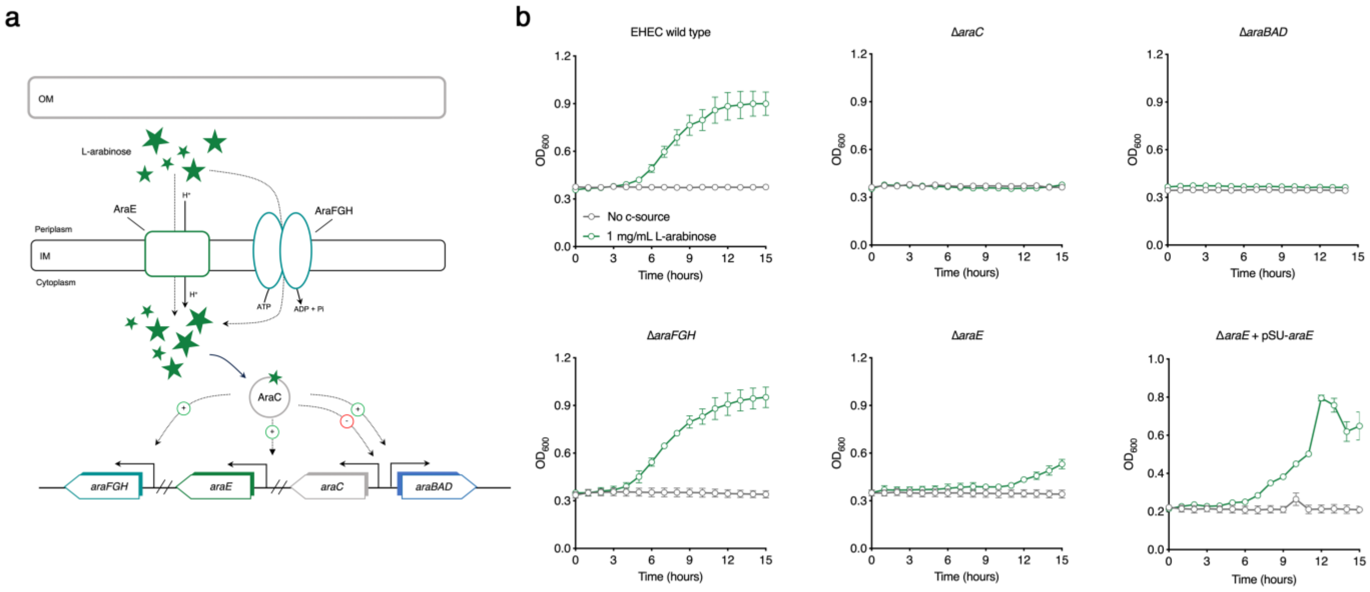

Supplementary Figure 8. Growth analysis of mutants from each stage of the ʟ-arabinose utilisation pathway in EHEC. a, Schematic illustration of the canonical pathway for ʟ-arabinose uptake, sensing and catabolism within the cell. **b,** Growth curves of wild type EHEC and the indicated deletion mutants in M9 minimal media with no carbon source (grey, control) or supplemented with 1 mg/ml ʟ-arabinose (green). Experiments Error bars represent the standard deviation derived from *n* = 3 biological replicates for all mutants except Δ*araBAD* (*n* = 2).

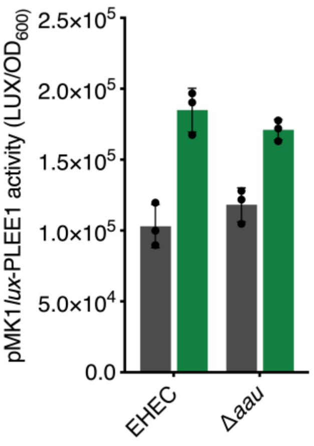

Supplementary Figure 9. Aau does not mediate enhanced LEE expression in response to L-arabinose *in vitro*. Transcriptional reporter assay of EHEC and the *aau* deletion mutant transformed with pMK1*lux*-PLEE1. Strains were cultured in MEM-HEPES alone (grey) or supplemented with L-arabinose (green). Data are depicted as luminescence units (LUX) divided by optical density (OD_{600}) sampled after 8 hours of growth. Error bars represent standard error of the mean derived from n = 3 biological replicates.

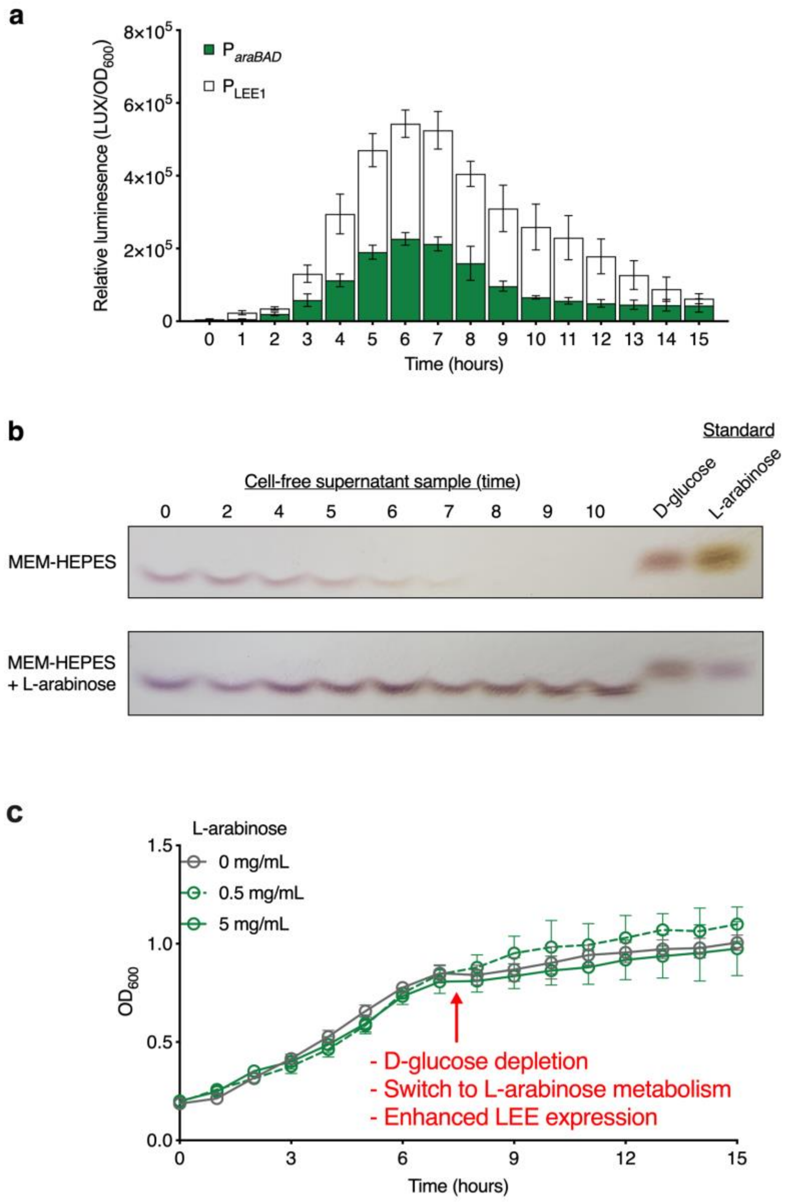

Supplementary Figure 10. ʟ-arabinose metabolism and enhanced LEE expression are co-ordinated in EHEC. a, Transcriptional reporter assay of EHEC transformed with either pMK1*lux*-P*LEE1* (white bars) or pMK1*lux*-P*araBAD* (green bars) cultured in MEM-HEPES supplemented with ʟ-arabinose. The experiments were performed under identical growth conditions and samples taken in parallel. The data are depicted as luminescence units (LUX) divided by optical density (OD_{600}) of the culture at each timepoint. Error bars indicate standard error of the mean from n = 3 (LEE1) or n = 5 (*araBAD*) biological replicates. **b,** Thin layer chromatography analysis of cell-free supernatant derived from cultures of EHEC grown in MEM-HEPES alone or supplemented with 1 mg/ml ʟ-arabinose. Samples were taken at the indicated timepoints and the standards for ᴅ-glucose and ʟ-arabinose are positioned on the right. The result represents a single replicate from three independent experiments. **c,** Growth curve of EHEC grown in MEM-HEPES alone or supplemented with various concentrations of ʟ-arabinose (n = 3). The red arrow indicates the point at which ᴅ-glucose is depleted from the media (derived from panel **b**) and the phenotypic switch associated with this event.

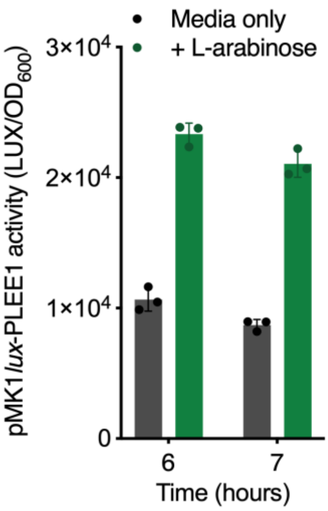

Supplementary Figure 11. ʟ-arabinose induces LEE expression in *C.rodentium.* Transcriptional reporter assay of wild type *C. rodentium* transformed with pMK1*lux*-PLEE1 cultured in MEM-HEPES alone (grey) or supplemented with ʟ-arabinose (green). Data are depicted as luminescence units (LUX) divided by optical density (OD_{600}) of the sample at various timepoints. Error bars represent standard error of the mean derived from n = 3 biological replicates.

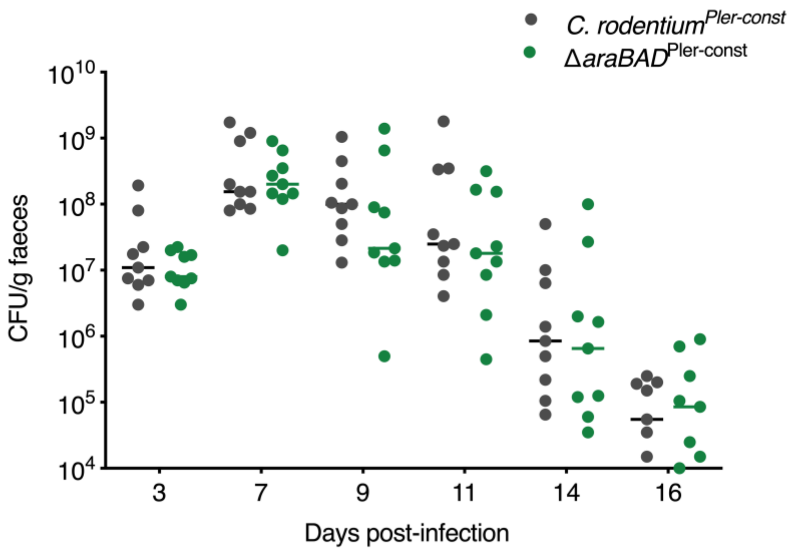

Supplementary Figure 12. Constitutive expression of *ler* overcomes the fitness defect associated with deletion of *araBAD*. Faecal shedding dynamics of BALB/c mice (n = 9) colonised with a 1:1 mixture of *C. rodentium*$^{\mathrm{P}\textit{ler}\text{-const}}$ (grey) and Δ*araBAD*$^{\mathrm{P}\textit{ler}\text{-const}}$ (green) by oral gavage. Data points indicate the relative CFU recovered from faecal samples on each day.

Supplementary Table 1 – Bacterial strains used in this study.

**Strain**	**Description**	**Reference**
ZAP193	EHEC O157:H7 str. ZAP193 (NCTC 12900) Stx^-	Roe *et al.* 2004
TUV93-0	EHEC O157:H7 str. EDL933 Stx^-	Campellone *et al*. 2002
Δ*araC*	TUV93-0 *araC* deletion mutant; Kan^R	This study
Δ*araBAD*	TUV93-0 *araBAD* deletion mutant; Kan^R	This study
Δ*araFGH*	TUV93-0 *araFGH* deletion mutant; Kan^R	This study
Δ*araE*	TUV93-0 *araE* deletion mutant; Cm^R	This study
Δ*rbsDACBKR*	TUV93-0 *rbsDACBKR* deletion mutant; Kan^R	This study
ZAP193 Δ*aau*	ZAP193 *Z0415-9* deletion mutant; Kan^R $Strep^R$	This study
ICC169	*C. rodentium* O152 serotype; Nal^R	Petty *et al.* 2010
ICC169 Δ*araBAD*	ICC169 *araBAD* deletion mutant; Kan^R	This study
ICC1370	*C. rodentium* constitutive-P_{ler} luminescence; Nal^R Kan^R	Mullineaux-Sanders *et al.* 2017
ICC1370 Δ*araBAD*	*ICC1370 ΔaraBAD* mutant; Nal^R Kan^R Cm^R	This study

Supplementary Table 2 – Plasmids used in this study.

**Plasmid**	**Description**	**Reference**
pMK1*lux*	pBR322 ori with the *luxCDABE* operon and MCS; Amp^R	Karavolos *et al.* 2008
pMK1*lux*-P_{LEE1}	pMK1*lux* with TUV93-0 LEE1 promoter cloned into MCS; Amp^R	This study
pMK1*lux*-P_{aau}	pMK1*lux* with *Z0415* promoter cloned into MCS; Amp^R	This study
pMK1*lux*-P_{araB}	pMK1*lux* with TUV93-0 *araB* promoter cloned into MCS; Amp^R	This study
pACYC184	p15A ori multicopy plasmid; Cm^R, Tet^R	Lab stock
pACYC184-*araC*	pACYC184 with TUV93-0 *araC* cloned into MCS; Cm^R, Tet^R	This study
pSUPROM	Cloning vector for expression under the TatA promoter; Kan^R	Jack *et al.* 2004
pSU-*araC*	pSUPROM with TUV93-0 *araC* cloned into MCS; Kan^R	This study
pSU-*araE*	pSUPROM with TUV93-0 *araE* cloned into MCS; Kan^R	This study
pSU-*araBADE*	pSUPROM with TUV93-0 *araBAD/araE* cloned into MCS; Kan^R	This study
pSU-*aau*	pSUPROM with ZAP193 *aau* cloned into MCS; Kan^R	This study
pKD46	LRed recombinase expressing plasmid; temperature sensitive; Amp^R	Datsenko and Wanner, 2000
pKD3	Template plasmid for LRed mutagenesis; Cm^R	Datsenko and Wanner, 2000
pKD4	Template plasmid for LRed mutagenesis; Kan^R	Datsenko and Wanner, 2000
pCP20	FLP recombinase expressing plasmid; temperature sensitive; Amp^R	Datsenko and Wanner, 2000
p*rpsM*:GFP	pACYC184, *rpsM*:GFP transcriptional fusion	Roe *et al.* 2004

Supplementary Table 3 – Primers used in this study.

Primer name	Description	Sequence
*Z0415-9*_LRed_Fwd	*Z0415-9* KO forward primer	gcgcgctaatttgggcgaacacttcctgactaccctgcaatgaggctgaagtgtaggctggagctgcttc
*Z0415-9*_LRed_Rev	*Z0415-9* KO reverse primer	cgcctgatatgtcatcgcggcaaaacgcgtccattgaatatagccaatatcatatgaatatcctccttag
*Z0415-9*_Check_Fwd	*Z0415-9* KO forward check primer	tctctccagcgcgctaat
*Z0415-9*_Check_Rev	*Z0415-9* KO reverse check primer	atgtcatcgcggcaaaac
EHEC_*araE*_LRed_Fwd	EHEC *araE* KO forward primer	attgttcacgtatttttcactatgtcttactctctgctggcaggaaaaagtgtaggctggagctgcttc
EHEC_*araE*_LRed_Rev	EHEC *araE* KO reverse primer	ctctattaacgaaaaaagggccggatgtacagcacatccggcccgtgaaacatatgaatatcctccttag
EHEC_*araE*_Check_Fwd	EHEC *araE* KO check forward primer	aatatccatcacataacggcatg
EHEC_*araE*_Check_Rev	EHEC *araE* KO check reverse primer	attcccagctcattcctccc
EHEC_*araFGH*_LRed_Fwd	EHEC *araFGH* KO forward primer	ttttgccctgcacaaaacgacactaaagctggagagaaccgtgtaggctggagctgcttc
EHEC_*araFGH*_LRed_Rev	EHEC *araFGH* KO reverse primer	tgtggtgggaaaaaacgttaaattgttgtggaaaaaagcacatatgaatatcctccttag
EHEC_*araFGH*_Check_Fwd	EHEC *araFGH* KO check forward primer	tcccgctaaatttatgcacgt
EHEC_*araFGH*_Check_Rev	EHEC *araFGH* KO check reverse primer	ttgcaacgaagaacagccaa
EHEC_*araBAD*_LRed_Fwd	EHEC *araBAD* KO forward primer	gcaactctctactgtttctccatacccgtttttttggatggagtgaaacggtgtaggctggagctgcttc
EHEC_*araBAD*_LRed_Rev	EHEC *araBAD* KO reverse primer	aaaaaaccaggcttgattatagcctggtttcatttgattggctgtggttttatacagtcacatatgaatatcctccttag
EHEC_*araBAD*_Check_Fwd	EHEC *araBAD* KO check forward primer	cgtcacactttgctatgcca
EHEC_*araBAD*_Check_Rev	EHEC *araBAD* KO check reverse primer	aagataaaacctgcctgcgc
EHEC_*araC*_LRed_Fwd	EHEC *araC* KO forward primer	tgcaatatggacaattggtttcttctctgaatggcgggagtatgaaaagtgtgtaggctggagctgcttc
EHEC_*araC*_LRed_Rev	EHEC *araC* KO reverse primer	caaaccctatgctactccgtcaagccgtcaattgtctgattcgttaccaacatatgaatatcctccttag
EHEC_*araC*_Check_Fwd	EHEC *araC* KO check forward primer	tcttctctgaatggcgggag

EHEC_*araC*_Check_Rev	EHEC *araC* KO check reverse primer	atggacgaagcagggattct
Crod_*araBAD*_LRed_Fwd	*C. rodentium araBAD* KO forward primer	cccactcactactgtttctccatac ccgtatttctggatggagtgaaac ggtgtaggctggagctgcttc
Crod_*araBAD*_LRed_Rev	*C. rodentium araBAD* KO reverse primer	tgtgttccggaataaaaatacgc gccactgtcgggacgcgtattttg catcatatgaatatcctccttag
Crod_*araBAD*_Check_Fwd	*C. rodentium araBAD* KO check forward primer	acaacggcagaaatgtccac
Crod_*araBAD*_Check_Rev	*C. rodentium araBAD* KO check reverse primer	ctttcattcgctggagggc
pMK1*lux*-P_{LEE1}_EHEC_Fwd	Forward primer for cloning EHEC LEE1 promoter with EcoRI	cccgaattcctgtaactcgaatta agt
pMK1*lux*-P_{LEE1}_EHEC_Rev	Reverse primer for cloning EHEC LEE1 promoter with BamHI	cccggatccaatctccgcatgctt taata
pMK1*lux*-P_{Z0415}_Fwd	Forward primer for cloning *Z0415* promoter with EcoRI	cccgaattcattcaccagaaatg gacg
pMK1*lux*-P_{Z0415}_Rev	Reverse primer for cloning *Z0415* promoter with BamHI	cccggatccatttcagcctcattg cag
pMK1*lux*-P_{araB}_Fwd	Forward primer for cloning *araB* promoter with EcoRI	cccgaattccgggaccaaagcca tgac
pMK1*lux*-P_{araB}_Rev	Reverse primer for cloning *araB* promoter with XbaI	gcgctctagacgtttcactccatc caaa
pMK1*lux*_Check_Fwd	Forward primer to check pMK1*lux* cloning	ctataaaaataggcgtatcac
pMK1*lux*_Check_Rev	Reverse primer to check pMK1*lux* cloning	ctggccgttaataatgaatg
pACYC184-*araC*_Fwd	*araC* Gibson assembly forward primer	tgaagtcagccccatacgattgc aatcgccatcgtttca
pACYC184-*araC*_Rev	*araC* Gibson assembly reverse primer	caatccatgccaacccgttcttat gacaacttgacggct
pACYC184_Check_Fwd	Forward primer to check pACYC184 cloning	gacgctcaaatcagtggtgg
pACYC184_Check_Rev	Reverse primer to check pACYC184 cloning	gcattcacagttctccgcaa
pACYC184_Linear_Fwd	pACYC184 linearisation forward primer	gaacgggttggcatggattg
pACYC184_Linear_Rev	pACYC184 linearisation reverse primer	atcgtatggggctgacttca
pSUPROM-*araC*_Fwd	Forward primer for cloning *araC* with BamHI	ggccggatcctcttctctgaatgg cgggag
pSUPROM-*araC*_Rev	Reverse primer for cloning *araC* with XbaI	ggcctctagaatggacgaagcag ggattct
pSUPROM-*araE*_Fwd	Forward primer for cloning *araE* with BamHI	ggccggatcctgtcttactctctgc tggca

pSUPROM-*araE*_Rev	Reverse primer for cloning *araE* with XbaI	ggcctctagaaacgagacaaac gcctcaac
pSUPROM-*araBADE*_F1_Fwd	*araBAD* fragment Gibson assembly forward primer	tctaccacagaggaggatccatg gcgattgcaattggc
pSUPROM-*araBADE*_F1_Rev	*araBAD* fragment Gibson assembly reverse primer	cagcagagagttactgcccgtaa tatgcc
pSUPROM-*araBADE*_F2_Fwd	*araE* fragment Gibson assembly forward primer	cgggcagtaactctctgctggcag gaaaaaatg
pSUPROM-*araBADE*_F2_Rev	*araE* fragment Gibson assembly reverse primer	ctcgaggggtcgactctagatca gacgccgatatttctcaac
pSUPROM-*aau*_F1_Fwd	*aau* Z0415-7 fragment Gibson assembly forward primer	tctaccacagaggaggatccatg atgaataaacgttttgttatc
pSUPROM-*aau*_F1_Rev	*aau* Z0415-7 fragment Gibson assembly reverse primer	ttttttcagttcagccatttaccac ctc
pSUPROM-*aau*_F2_Fwd	*aau* Z0418-9 fragment Gibson assembly forward primer	aaatggctgaactgaaaaaacgc catgaattc
pSUPROM-*aau*_F2_Rev	*aau* Z0418-9 fragment Gibson assembly reverse primer	ctcgaggggtcgactctagattaa accacccgatccag
pSUPROM_Check_Fwd	Forward primer to check pSUPROM cloning	ctcttcgctattacgccagc
pSUPROM_Check_Rev	Reverse primer to check pSUPROM cloning	accctcatcagtgccaacat
pSUPROM_Linear_Fwd	pSUPROM linearisation forward primer	tctagactcgacccctcg
pSUPROM_Linear_Rev	pSUPROM linearisation reverse primer	ggatcctcctctgtggtag
*escT*_Fwd	*escT* RT-qPCR forward primer	tttgggctatagatgcggct
*escT*_Rev	*escT* RT-qPCR reverse primer	ggatgaatcgcttatagacggg
*escC*_Fwd	*escC* RT-qPCR forward primer	gctgaagtgagtgctcgttt
*escC*_Rev	*escC* RT-qPCR reverse primer	cctcaagcgggtcaataacg
*escV*_Fwd	*escV* RT-qPCR forward primer	ctaaaagttctccagtacgtgc
*escV*_Rev	*escV* RT-qPCR reverse primer	tcgccagagaaatcatcattca
*espA*_Fwd	*espA* RT-qPCR forward primer	ttcctgtaaatccgatgcgc
*espA*_Rev	*espA* RT-qPCR reverse primer	tggttgacgctttagatgcc
*tir*_Fwd	*tir* RT-qPCR forward primer	ttcctgtaaatccgatgcgc
*tir*_Rev	*tir* RT-qPCR reverse primer	atcgagcggaccatgatcat
*Z0415*_Fwd	*Z0415* RT-qPCR forward primer	tggtgtcttcgctgttattagg
*Z0415*_Rev	*Z0415* RT-qPCR reverse primer	cacggcataccatcgacttta
*Z0417*_Fwd	*Z0417* RT-qPCR forward primer	tggaagtttccgaccgtattt
*Z0417*_Rev	*Z0417* RT-qPCR reverse primer	tcatcaggaaaccgagttgtt
*Z0418*_Fwd	*Z0418* RT-qPCR forward primer	gccttactggttaatcgcctac
*Z0418*_Rev	*Z0418* RT-qPCR reverse primer	gtacagccacacacctttactc
Housekeeping_GroEL_Fwd	GroEL RT-qPCR forward primer	accgctgcagttgaagaa
Housekeeping_GroEL_Rev	GroEL RT-qPCR reverse primer	ctacggtttcgtcggagttag
Housekeeping_GapA_Fwd	GapA RT-qPCR forward primer	cggtaccgttgaagtgaaaga
Housekeeping_GapA_Rev	GapA RT-qPCR reverse primer	acttcgtcccatttcaggttag

Supplementary references:

Campellone, K.G., Giese, A., Tipper, D.J., and Leong, J.M. A tyrosine- phosphorylated 12-amino-acid sequence of enteropathogenic *Escherichia coli* Tir binds the host adaptor protein Nck and is required for Nck localization to actin pedestals. *Molecular Microbiology* **43***,* 1227–1241 (2002).

Datsenko, K. A. and Wanner, B. L. One-step inactivation of chromosomal genes in *Escherichia coli* K-12 using PCR products. *Proceedings of the National Academy of Sciences U. S. A.* **97**, 6640–6645 (2000).

Jack, R. L. *et al.* Coordinating assembly and export of complex bacterial proteins. *EMBO Journal* **23**, 3962–3972 (2004).

Karavolos, M. H. *et al.* Adrenaline modulates the global transcriptional profile of *Salmonella* revealing a role in the antimicrobial peptide and oxidative stress resistance responses. *BMC Genomics* **6**, 458 (2008).

Mullineaux-Sanders, C. *et al*. *Citrobacter rodentium* relies on commensals for colonization of the colonic mucosa. *Cell Reports* **21**, 3381–3389 (2017).

Petty, N. K. *et al*. The *Citrobacter rodentium* genome sequence reveals convergent evolution with human pathogenic *Escherichia coli*. *Journal of Bacteriology* **192**, 525–538 (2010).

Roe AJ, *et al*. Co-ordinate single-cell expression of *LEE4*- and *LEE5*-encoded proteins of *Escherichia coli* O157:H7. *Molecular Microbiology* **54**, 337–352 (2004).

Uncropped images:

Supplementary Figure 10b – Full TLC results

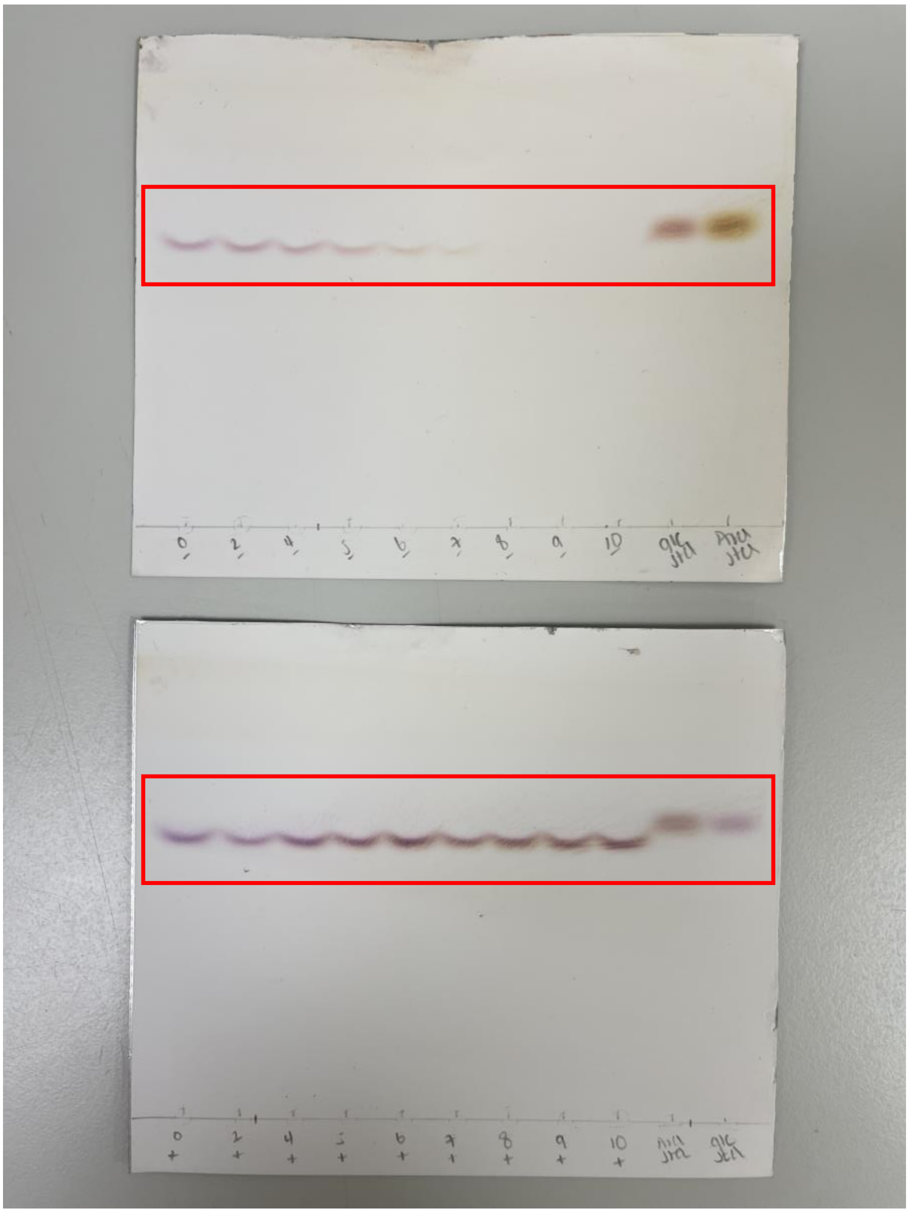

Supplement: Supplementary file 1 — Supplementary Information [file 41467_2024_48933_MOESM1_ESM.pdf]
