# Supplementary material for: Metabolism of l-arabinose converges with virulence regulation to promote enteric pathogen fitness
Source: Nat Commun. 2024 May 25;15:4462. doi: 10.1038/s41467-024-48933-7 (PMC11127945; doi:10.1038/s41467-024-48933-7)
Supplement: Supplementary file 3 — Description of Additional Supplementary Files [file 41467_2024_48933_MOESM3_ESM.pdf]

Description of Additional Supplementary files

Supplementary Data 1 – Summary of differentially expressed genes identified by RNA-seq. Data derived from EHEC TUV93-0 cultures grown in MEM-HEPES alone (control) or supplemented with 1 mg/ml L-arabinose (treatment).
