# Supplementary material for: Metabolism of l-arabinose converges with virulence regulation to promote enteric pathogen fitness
Source: Nat Commun. 2024 May 25;15:4462. doi: 10.1038/s41467-024-48933-7 (PMC11127945; doi:10.1038/s41467-024-48933-7)
Supplement: Supplementary file 5 — Reporting Summary [file 41467_2024_48933_MOESM5_ESM.pdf]

nature portfolio

Corresponding author(s): James Connolly

Last updated by author(s): May 4, 2024

Reporting Summary

Nature Portfolio wishes to improve the reproducibility of the work that we publish. This form provides structure for consistency and transparency in reporting. For further information on Nature Portfolio policies, see our Editorial Policies and the Editorial Policy Checklist.

Statistics

For all statistical analyses, confirm that the following items are present in the figure legend, table legend, main text, or Methods section.

n/a	Confirmed	
☐	☒	The exact sample size (*n*) for each experimental group/condition, given as a discrete number and unit of measurement
☐	☒	A statement on whether measurements were taken from distinct samples or whether the same sample was measured repeatedly
☐	☒	The statistical test(s) used AND whether they are one- or two-sided<br>*Only common tests should be described solely by name; describe more complex techniques in the Methods section.*
☐	☒	A description of all covariates tested
☐	☒	A description of any assumptions or corrections, such as tests of normality and adjustment for multiple comparisons
☐	☒	A full description of the statistical parameters including central tendency (e.g. means) or other basic estimates (e.g. regression coefficient) AND variation (e.g. standard deviation) or associated estimates of uncertainty (e.g. confidence intervals)
☐	☒	For null hypothesis testing, the test statistic (e.g. *F*, *t*, *r*) with confidence intervals, effect sizes, degrees of freedom and *P* value noted<br>*Give P values as exact values whenever suitable.*
☒	☐	For Bayesian analysis, information on the choice of priors and Markov chain Monte Carlo settings
☐	☒	For hierarchical and complex designs, identification of the appropriate level for tests and full reporting of outcomes
☒	☐	Estimates of effect sizes (e.g. Cohen's *d*, Pearson's *r*), indicating how they were calculated

Our web collection on statistics for biologists contains articles on many of the points above.

Software and code

Policy information about availability of computer code

Data collection	Please methods section entitled. Raw data was generated using the Illumina Next-seq platform. Genome sequence data were retrieved from the National Centre for Biotechnology Information (NCBI) Sequence Read Archive using the 'prefetch' and 'fastq-dump' tools within the SRA Toolkit v2.9.0-mac64.
Data analysis	Please methods section entitled. Raw data were analysed using SALMON. Data was normalised and differential expression calculated using DESeq2 v.1.28.1. Volcano plots were generated using R studio enhanced volcano plot package v.1.6.0. The raw data has been deposited in the European Nucleotide Archive under the accession number XXX. Software packages used for gene-carriage analysis was as follows: FastQC v0.11.8; MultiQC v1.11; Trimmomatic v0.36; ART-MountRainier-2016-06-05; Burrows–Wheeler Aligner v0.7.15; SAMtools v1.2; Picard v2.7.1; Genome Analysis Tool Kit v3.2-2; BEDTools v2.18.2; SNPEff v4.1; SPANDx v3.2; Shovill v1.0.4; Seqtk v1.3-r106; Lighter v1.1.2; FLASH v1.2.11; SPAdes v3.13.1; Samclip v0.2; SAMtools v1.8; BWA-MEM v0.7.17-r1188; Pilon v1.22; QUAST v4.5; MLST v2.19.0; RAxML v8.2.10; FigTree v1.4.4. Protein bioinformatics were performed using MEGAX v10.1.8, MUSCLE and AlphaFold v2.1.1.

For manuscripts utilizing custom algorithms or software that are central to the research but not yet described in published literature, software must be made available to editors and reviewers. We strongly encourage code deposition in a community repository (e.g. GitHub). See the Nature Portfolio guidelines for submitting code & software for further information.

Data

Policy information about availability of data

All manuscripts must include a data availability statement. This statement should provide the following information, where applicable:

- Accession codes, unique identifiers, or web links for publicly available datasets
- A description of any restrictions on data availability
- For clinical datasets or third party data, please ensure that the statement adheres to our policy

The transcriptomic sequencing data is available from the NCBI Gene Expression Omnibus under the accession number GSE262155. The source data for Figs. 2-6, Supplementary Figs. 3-5 and 7-12 are provided as a Source Data File. Publicly available genome sequences were obtained from the NCBI Sequence Read Archive.

Research involving human participants, their data, or biological material

Policy information about studies with human participants or human data. See also policy information about sex, gender (identity/presentation), and sexual orientation and race, ethnicity and racism.

Reporting on sex and gender	N/A
Reporting on race, ethnicity, or other socially relevant groupings	N/A
Population characteristics	N/A
Recruitment	N/A
Ethics oversight	N/A

Note that full information on the approval of the study protocol must also be provided in the manuscript.

Field-specific reporting

Please select the one below that is the best fit for your research. If you are not sure, read the appropriate sections before making your selection.

☒ Life sciences ☐ Behavioural & social sciences ☐ Ecological, evolutionary & environmental sciences

For a reference copy of the document with all sections, see nature.com/documents/nr-reporting-summary-flat.pdf

Life sciences study design

All studies must disclose on these points even when the disclosure is negative.

Sample size	Sample sizes for in vivo studies were determined using the G*power tool (Faul et al., Behavior Research Methods 2007) based on our own pilot data and the extensive literature utilizing the Citrobacter rodentium and Escherichia coli Streptomycin mouse models, with the appropriate statistical analyses described and performed.
Data exclusions	We did not need to exclude any data.
Replication	All in vivo experiments were performed in two independent cohorts. All in vitro, bacterial and cell infection experiments were performed in biological triplicate on separate occasions (with technical replicates where appropriate described in the methods). All attempts to replicate the data were successful. RNA-seq data was determined from three independent repeats.
Randomization	Mice were assigned experimental groups at random to different cages and on different occasions to avoid batch or cage effects.
Blinding	Bacterial inocula were prepared by one team member and administered by another. Bacterial burdens were determined blindly by one team member gathering samples and another analysing the data. Mice were health scored blindly.

Reporting for specific materials, systems and methods

We require information from authors about some types of materials, experimental systems and methods used in many studies. Here, indicate whether each material, system or method listed is relevant to your study. If you are not sure if a list item applies to your research, read the appropriate section before selecting a response.

Materials & experimental systems

n/a	Involved in the study	
☐	☒	Antibodies
☐	☒	Eukaryotic cell lines
☒	☐	Palaeontology and archaeology
☐	☒	Animals and other organisms
☒	☐	Clinical data
☒	☐	Dual use research of concern
☒	☐	Plants

Methods

n/a	Involved in the study	
☒	☐	ChIP-seq
☒	☐	Flow cytometry
☒	☐	MRI-based neuroimaging

Antibodies

Antibodies used	Anti-EspD antibodies are polyclonal and were generated in mice from recombinantly purified proteins. They were verified for cross-reactivity by testing against an isogenic espD deletion background. These antibodies are not commercially available. Anti-GroEL antibodies are polyclonal and were generated in rabbits. These are commercial antibodies and were purchased from Abcam (ab90522).
Validation	Validation of these antibodies was performed using isogenic gene deletion strains to test for non-specific cross reactivity. For commercial antibodies see product information sheet from the suppliers website. Results using these antibodies have been previously published (ISME J 9:1039-1051; PLoS Path 12:e1005359; Mol Microbiol 105:606-619).

Eukaryotic cell lines

Policy information about cell lines and Sex and Gender in Research

Cell line source(s)	HeLa cells (ATCC)
Authentication	Extensively used in similar studies and published by ourselves and many other research groups.
Mycoplasma contamination	All PCR screening for contamination tested negative.
Commonly misidentified lines (See ICLAC register)	N/A

Animals and other research organisms

Policy information about studies involving animals; ARRIVE guidelines recommended for reporting animal research, and Sex and Gender in Research

Laboratory animals	Mice. BALB/c female, aged 7-8 weeks old.
Wild animals	N/A
Reporting on sex	Single sex experiments were performed to assist randomisation and to allow comparison with extensively published literature using the same described sex.
Field-collected samples	N/A
Ethics oversight	All animal experiments were performed in strict accordance with the United Kingdom Home Office Animals Scientific Procedures Act of 1986 under the personal project licence number PP8850146. The experiments were subject to local ethical approval and consideration given to the refine, reduce and replace principals wherever possible so as all efforts were made to minimize animal suffering.

Note that full information on the approval of the study protocol must also be provided in the manuscript.

Plants

Seed stocks	N/A
Novel plant genotypes	N/A
Authentication	N/A
